# A Human Platelet Receptor Protein Microarray Identifies the High Affinity Immunoglobulin E Receptor Subunit α (FcεR1α) as an Activating Platelet Endothelium Aggregation Receptor 1 (PEAR1) Ligand* [Fn FN2]

**DOI:** 10.1074/mcp.M114.046946

**Published:** 2015-02-23

**Authors:** Yi Sun, Christophe Vandenbriele, Alexandre Kauskot, Peter Verhamme, Marc F. Hoylaerts, Gavin J. Wright

**Affiliations:** From the ‡Cell Surface Signalling Laboratory, Wellcome Trust Sanger Institute, Cambridge, CB10 1SA, United Kingdom and; §Center for Molecular and Vascular Biology, Department of Cardiovascular Sciences, University of Leuven, Leuven 3000, Belgium

## Abstract

Genome-wide association studies to identify loci responsible for platelet function and cardiovascular disease susceptibility have repeatedly identified polymorphisms linked to a gene encoding platelet endothelium aggregation receptor 1 (PEAR1), an “orphan” cell surface receptor that is activated to stabilize platelet aggregates. To investigate how PEAR1 signaling is initiated, we sought to identify its extracellular ligand by creating a protein microarray representing the secretome and receptor repertoire of the human platelet. Using an avid soluble recombinant PEAR1 protein and a systematic screening assay designed to detect extracellular interactions, we identified the high affinity immunoglobulin E (IgE) receptor subunit α (FcεR1α) as a PEAR1 ligand. FcεR1α and PEAR1 directly interacted through their membrane-proximal Ig-like and 13th epidermal growth factor domains with a relatively strong affinity (*K_D_* ∼ 30 nm). Precomplexing FcεR1α with IgE potently inhibited the FcεR1α-PEAR1 interaction, and this was relieved by the anti-IgE therapeutic omalizumab. Oligomerized FcεR1α potentiated platelet aggregation and led to PEAR1 phosphorylation, an effect that was also inhibited by IgE. These findings demonstrate how a protein microarray resource can be used to gain important insight into the function of platelet receptors and provide a mechanistic basis for the initiation of PEAR1 signaling in platelet aggregation.

Platelets play a vital role in preserving blood circulation in response to vessel injury by detecting lesions, aggregating to form a hemostatic plug, and nucleating the formation of a fibrin-rich, injury-occluding clot. Although necessary to prevent blood loss at sites of tissue trauma, clot formation must also be attenuated to prevent blockage of the vasculature serving vital organs that would cause life-threatening ischemia and infarction. Inappropriate platelet aggregation and vessel occlusion, often triggered by atherosclerotic plaque rupture, is a major pathological process that is a major contributor to cardiovascular disease, which is the leading cause of mortality worldwide (1). With the eventual aim of guiding the development of new treatments and diagnostic assays, genome-wide association studies using large patient cohorts have identified several genetic loci that are associated with cardiovascular disease susceptibility and platelet function (2, 3). Among the candidate genes identified, polymorphisms linked to *PEAR1* have been repeatedly linked to natural variation in response to platelet agonists in several independent studies (3–7). *PEAR1* encodes platelet endothelium activation receptor 1 (PEAR1;^1^ also known as multiple epidermal growth factor-like domain protein 12 (MEGF12) or JEDI-1), a platelet cell surface receptor that was originally identified as a protein phosphorylated in response to platelet aggregation (8, 9). PEAR1 is expressed at low levels on the surface of circulating platelets but is significantly up-regulated during platelet activation when released from cytoplasmic α-granules (8). Consistent with polymorphisms linked to *PEAR1* being associated with cardiovascular disease and platelet function, PEAR1-mediated signaling was shown to reinforce and stabilize the interactions between platelets within a forming aggregate (8). PEAR1 is an orphan receptor, and an important unanswered question in understanding the mechanism of PEAR1 function during platelet aggregation, therefore, is the identification of its activating ligand.

Identifying interactions between membrane-embedded receptor proteins is technically challenging, and many commonly used approaches such as biochemical purifications are generally not suitable to detect them. This is largely due to the amphipathic nature of membrane-embedded proteins that makes them difficult to solubilize in detergents that retain their native conformation and the fact that their extracellular interactions are often highly transient, having half-lives of just fractions of a second (10). To address these issues, we and others have developed assays based on detecting direct protein interactions between the entire ectodomains of cell surface receptors expressed as soluble recombinant proteins in eukaryotic cells (11–14). Using this approach, binding avidity can be increased by the purposeful inclusion of oligomerizing tags to overcome the fleeting nature of these interactions. In our assay, avidity-based extracellular interaction screen (AVEXIS), arrays of monomeric biotinylated “bait” proteins are screened against multimerized, enzyme-tagged, highly avid “preys” (11, 15); a schematic of the assay is shown in supplemental Fig. S1. The likelihood that the extracellular binding functions of receptors are preserved is increased by expressing whole ectodomains in mammalian cells so that structurally critical posttranslational modifications such as disulfide bonds are faithfully added. Consequently, this method has identified interactions that have subsequently been demonstrated to be essential for cellular recognition processes *in vivo* (16–18).

In this study, we have compiled a protein resource representing the cell surface receptor repertoire and secretome of the human platelet that will be useful to identify intercellular interactions important for platelet biology. As an example, we identify the activating ligand for PEAR1 as the high affinity immunoglobulin E (IgE) receptor subunit α (FcεR1α) and show that multimerized FcεR1α potentiated platelet aggregation and led to PEAR1 phosphorylation, an effect that was specifically inhibited by IgE.

## EXPERIMENTAL PROCEDURES

### 

#### 

##### Human Platelet Protein Selection and Expression Plasmid Construction

After compiling a list of platelet receptor proteins and classifying them into structural categories, the extent of each ectodomain was identified by careful manual examination of its structural features such as signal peptides, transmembrane regions, and glycosylphosphatidylinositol anchors; for secreted proteins, the entire protein was used. The ectodomain regions were codon-optimized for human expression, chemically synthesized with unique flanking NotI (5′) and AscI (3′) restriction sites (GeneArt AG), and subcloned into bait expression plasmids according to its structural class. Although we have previously expressed type I/glycosylphosphatidylinositol-anchored and secreted proteins for interaction screening (11), we designed new expression vectors to express the ectodomains of type II and multispan transmembrane proteins in a way that would most appropriately preserve their structure when presented at the cell surface *in vivo*. Similarly, for multimeric protein complexes such as integrins, glycoprotein 1bαβ, and fibrinogen, care was taken to design constructs that would promote correct and active complex formation. The truncated fragments of PEAR1 and FcεR1α were designed based on their domain structures, amplified by PCR from the full-length constructs, and cloned into an expression vector containing an exogenous signal peptide and C-terminal His_6_ and biotinylatable peptide sequence. The 218- and 325-amino acid fragments encoding the Cε3–4 and Cε2–4 domains of the IgE constant heavy chain were amplified from plasmid pFUSE-CHIg-hE (Invivogen) and cloned into a vector containing an exogenous signal peptide and C-terminal His_6_ tag. IgE fragments were expressed and purified as described below. All plasmid constructs are openly available from Addgene.

##### Recombinant Protein Expression

All proteins were produced by transient transfection using human embryonic kidney HEK293E cells as described (11) to ensure posttranslational modifications such as disulfide bonds and glycans were added. Proteins were purified using their His_6_ tag using a bespoke supernatant loading rig and 96-well Ni^2+^-nitrilotriacetic acid filter plates (15). Heat-labile immunoreactivity to demonstrate folding was confirmed by heat-denaturing the proteins for 10 min at 90 °C before capture on a streptavidin-coated plate via their biotin tag and determination of immunoreactivity by ELISA as described (19).

##### Construction of the Human Platelet Receptor Protein Microarray

Normalized bait proteins were diluted in phosphate-buffered saline (PBS) supplemented with 50% glycerol, 0.02% Tween, and 0.5% bovine serum albumin (BSA) prior to printing. Bait proteins were printed on streptavidin-coated slides that also contained an inert hydrogel coating (XanTec) using a Marathon arrayer (Arrayjet) at 60% relative humidity according to the manufacturer's instructions. Printed slides were incubated for 1 h at 60% relative humidity and blocked with PBS containing 1% BSA and 10 mm d-biotin for 45 min. Slides were then incubated with normalized prey proteins for 1 h before being incubated with an anti-FLAG-horseradish peroxidase antibody (Sigma; 1:1000) for 1 h and finally detected by tyramide signal amplification Alexa Fluor 555 substrate (Invitrogen) for 1 h. Between different incubation steps, slides were washed three times in PBS buffer containing 0.1% Tween 20 with gentle rocking. Arraying, incubations, and washing steps were performed at 22 °C. Positive interactions were identified and quantified by scanning slides with a ScanArray Express microarray scanner (PerkinElmer Life Sciences) at 550 nm.

##### AVEXIS Interaction Screening

To determine the effect of IgE/omalizumab and map the interacting domains of the FcεR1α-PEAR1 interaction, we used the AVEXIS method formatted on streptavidin-coated 96-well microtiter plates as described (11, 17). Briefly, bait and prey proteins were first normalized to activities suitable for the AVEXIS assay (20). Biotinylated baits that had been either purified or dialyzed against HEPES-buffered saline were immobilized in streptavidin-coated 96-well microtiter plates (Nunc). Preys were incubated for 2 h and washed three times with PBS and 0.1% Tween 20 and once in PBS, 125 μg/ml nitrocefin was added, and absorbance values were measured at 485 nm on a Pherastar Plus (BMG Laboratories). A protein consisting of the Cd4d3+4 tag alone was used as a negative control bait, and a biotinylated anti-Cd4 monoclonal antibody (anti-prey) was used as a positive control as required.

##### Surface Plasmon Resonance Studies

All surface plasmon resonance studies were performed on a Biacore T100 instrument essentially as described (17). Purified analyte proteins were resolved by gel filtration just prior to use in surface plasmon resonance experiments. Increasing concentrations of proteins were injected at 10 μl/min for equilibrium analysis or high flow rates (100 μl/min) for kinetic studies to minimize the confounding effects of analyte rebinding. Both kinetic and equilibrium binding data were analyzed using the manufacturer's Biacore T100 evaluation software (Biacore).

##### Platelet Aggregometry Assays

Venous blood was collected from normal donors following guidelines from the ethics committee of the Leuven University Hospital (number B322201111373/S53239). Platelet-rich plasma was prepared, and platelet aggregation was monitored as described (8). Platelets (4 × 10^5^/μl) were preincubated with either soluble recombinant purified pentameric proteins extensively dialyzed into PBS (PEAR1 (s5-PEAR1), FcεR1α (s5-FcεR1α), and control rat Cd200 all used at 10 μg/ml) or the commercially available antibodies human IgE (10 μg/ml (53 nm); Abcam), anti-PEAR1 (3 μg/ml; R&D Systems), and omalizumab (10 μg/ml (67 nm); Xolair®, Novartis) at 37 °C as appropriate. The IgE fragments were used at an equimolar concentration of 67 nm. Platelet aggregation was triggered by collagen with the concentration adjusted to reach 40–70% platelet aggregation for each donor and measured as the percentage of change in light transmission relative to a blank (buffer without platelets) set to 100%.

##### Western Blotting

Western blotting was performed essentially as described (8) using the primary antibodies PEAR1-EC antibody (R&D Systems), PEAR1-EC antibody (Santa Cruz Biotechnology), FcεR1α antibody (LifeSpan Biosciences), anti-PLCγ2 (Sigma), anti-PLCγ2-P (Cell Signaling Technology), anti-AKT-P (Cell Signaling Technology), and anti-phosphoprotein (Tyr(P)) 4G10 Platinum (Millipore). After adding horseradish peroxidase-conjugated secondary antibodies (Dako), immunoreactive bands were visualized by ECL (Amersham Biosciences). PEAR1 phosphorylation on Tyr residues, referred to as PEAR1-P, was evaluated after immunoprecipitation with PEAR1-EC antibody and detection of Tyr(P) by 4G10 platinum as previously described (8).

##### Platelet Immunocytochemistry

Platelets were stained essentially as described (8) using the primary antibodies anti-human IgE (5 μg/ml; BD Pharmingen), anti-human FcεRI (5 μg/ml; eBioscience), and anti-human PEAR1 (2 μg/ml; R&D Systems); washed; and detected using an appropriate Alexa Fluor 488-conjugated secondary antibody (1:200; Invitrogen) for 60 min at 37 °C. Coverslips were mounted with 4′,6-diamidino-2-phenylindole ProLong Gold (Invitrogen), sealed on glass slides, and analyzed using a Zeiss ELYRA superresolution microscope and Zen 2011 image software (Carl Zeiss).

## RESULTS

### 

#### 

##### A Protein Resource Representing the Secretome and Receptor Repertoire of the Human Platelet

**Fig. 1.**
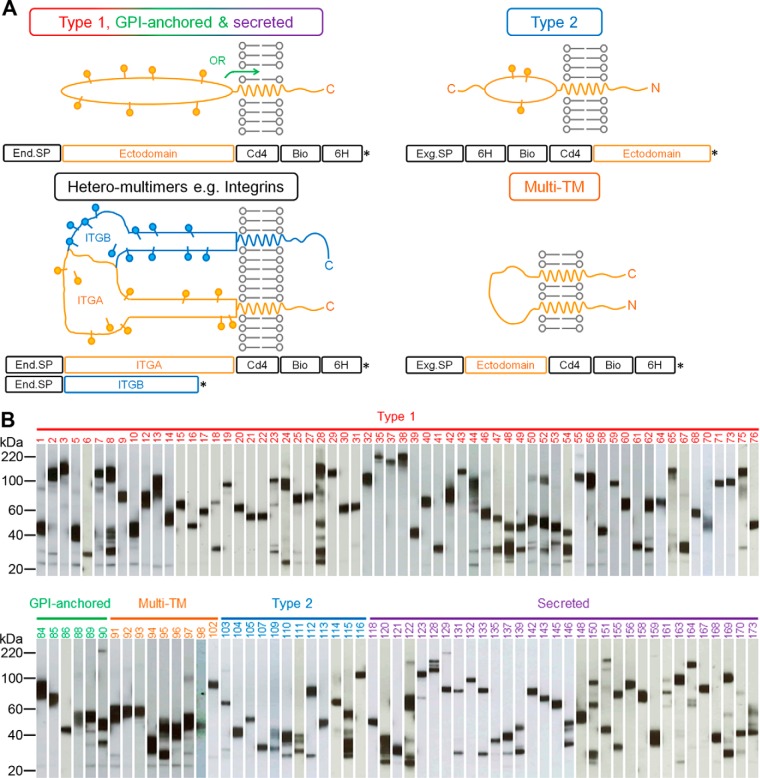
**Design and expression of a human platelet receptor library for common structural classes of cell surface and secreted proteins for AVEXIS.** *A*, schematics show the design of ectodomain expression constructs. Type II and multispan proteins contained an exogenous signal peptide (*Exg.SP*), whereas the others retain their endogenous signal peptide (*End.SP*). Heteromeric complexes such as the integrins shown here were tagged on only one chain to ensure expression of only tagged complexes. *, stop codon. *B*, an anti-biotin Western blot of the 121 bait proteins organized into their structural categories. The majority of proteins were expressed at the expected size with little processing. Numbering is according to supplemental Table S2; note that the five expressed heteromeric complexes are not included because only one chain would have been detected. *GPI*, glycosylphosphatidylinositol; *Bio*, biotin; *6H*, His_6_; *ITG*, integrin; *TM*, transmembrane.

To identify an activating ligand for PEAR1, we first created a protein library that represented the cell surface receptor repertoire and secretome of the human platelet expressed as secreted recombinant proteins. We and others have previously only expressed type I/glycosylphosphatidylinositol-anchored and secreted proteins for large scale extracellular interaction screening (11–14), but because platelets also express cell surface proteins from other structural classes, we designed new expression plasmids. We constructed these new expression plasmids with the goal of preserving the structure of the receptor when displayed at the platelet surface (Fig. 1*A*). In the case of multimeric protein complexes such as integrins, glycoprotein 1bαβ, and fibrinogen, care was taken to design the constructs such that they would promote correct and active complex formation; for example, secretion of integrin α chains can be dependent on the presence of the β chain (21), and so tags were only added to the α chain, ensuring the expression of a tagged α/β complex in an active conformation (22) (Fig. 1*A*). We next compiled a list of secreted and membrane proteins expressed by human platelets from 18 proteomic data sets and the literature (supplemental Table S1). To increase the chances of identifying functionally relevant interactions, each protein was classified into one of six structural categories. The entire extent of the ectodomain region was then determined, and an expression construct was manually designed by pairing it with an appropriate expression vector. The final library contained 173 proteins and complexes (76 type I, seven glycosylphosphatidylinositol-anchored, 14 type II, 57 secreted, seven heterodimeric complexes, and 12 multispan transmembrane proteins) represented by a total of 178 plasmids (supplemental Table S2). The proteins were expressed and purified as monomeric biotinylated “baits” in mammalian cells so that structurally important posttranslational modifications such as disulfide bonds and glycans were added. Similar to other secreted recombinant protein libraries that we (11, 17, 23, 24) and others (13, 25, 26) have made, expression levels varied widely but averaged ∼3 μg/ml (supplemental Table S2). After purification, 126 proteins were expressed at sufficient levels for our interaction screens. As expected, Western blots of the protein library showed that the vast majority (113 of 121; 93%) of proteins exhibited mass heterogeneity centered on their expected size, suggesting the presence of different glycoforms (Fig. 1*B*). The recombinant proteins were antigenically active as assessed by demonstrating heat-labile immunoreactivity to a panel of monoclonal antibodies recognizing at least one protein from each structural category (supplemental Fig. S2A). The library of biotinylated bait proteins was serially diluted and arrayed on streptavidin-coated glass slides (supplemental Fig. S3). Proteins immobilized on the slides retained their immunoreactivity to monoclonal antibodies known to stain native proteins at the cell surface (supplemental Fig. S2B). These plasmids and recombinant protein library represent a valuable resource for the investigation of human platelet biology particularly in regard to the role of these proteins in thrombosis.

##### Systematic Interaction Screening of the Platelet Receptor Microarray Identified FcεR1α as a Ligand for PEAR1

**Fig. 2.**
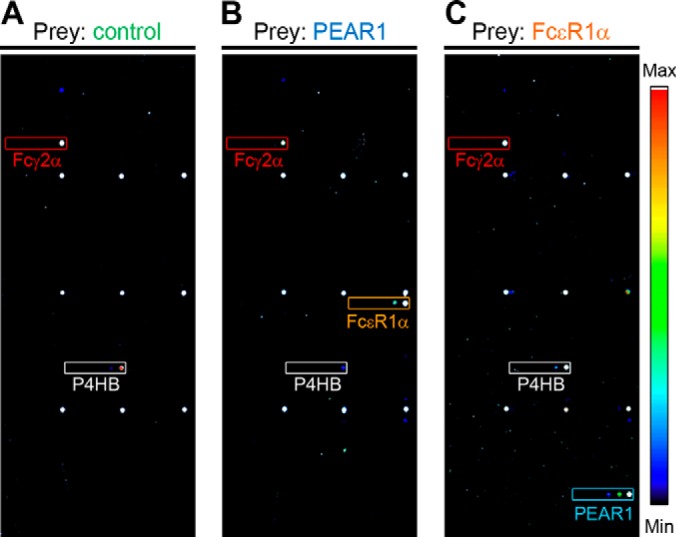
**A human platelet secretome and receptor protein microarray identifies FcεR1α as a ligand for PEAR1.** Soluble recombinant biotinylated proteins representing the secretome and receptor repertoire of the human platelet were purified and arrayed in six 3-fold dilutions on streptavidin-coated slides. *A*, the array was screened with a control (rat Cd200) pentamerized FLAG-tagged prey that bound the background baits FcγR2α (*red box*) and P4HB (*white box*). *B*, PEAR1 prey additionally interacted with the FcεR1α bait (*orange box*) when compared with the control in *A*; fluorescence intensities are quantified in supplemental Fig. S4. *C*, FcεR1α prey additionally interacted with the PEAR1 bait (*blue box*) in comparison with the control. Note that the nine regularly spaced markers are orientation markers and that the *boxed* areas marked on the array enclose the location of the six spots containing the dilutions of the named immobilized baits (see supplemental Fig. S3).

To identify an activating ligand for PEAR1 during platelet aggregation, we systematically screened the human platelet receptor microarray using the AVEXIS assay, which required expressing the entire ectodomain of PEAR1 as a recombinant pentameric, FLAG-tagged soluble “prey.” Probing the array with a control pentameric FLAG-tagged prey protein labeled the high affinity IgG receptor Fcγ2α (which directly bound the anti-FLAG antibody) and P4HB, a bait that presumably interacted with the tags on the control prey (Fig. 2*A*). Screening the receptor microarray with the PEAR1 prey identified the high affinity IgE-binding subunit FcεR1α as a PEAR1 ligand (Fig. 2*B* and supplemental Fig. S4). Because PEAR1 was presented as a bait on the microarray, to rule out any possible artifacts, we expressed the entire ectodomain of FcεR1α as a prey protein and rescreened the array. As expected, we observed that the FcεR1α prey interacted with the PEAR1 bait, showing that we could detect the same interaction but in the reciprocal bait-prey orientation (Fig. 2*C*).

##### PEAR1 and FcεR1α Directly Interact through Their Membrane-proximal Domains

**Fig. 3.**
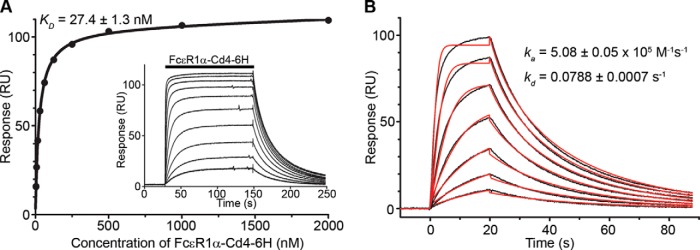
**FcεR1α and PEAR1 directly and specifically interact with a relatively high affinity.** *A*, purified monomeric FcεR1α-Cd4-His_6_ was serially diluted and injected over immobilized PEAR1 until equilibrium was achieved (*inset*). Binding data that had been reference-subtracted were plotted as a binding curve, and a *K_D_* of 27.4 ± 1.3 nm was calculated. *B*, association and dissociation rate constants derived from an independent kinetic analysis of the FcεR1α-PEAR1 interaction were consistent with the equilibrium analysis. Seven serial dilutions of purified, soluble FcεR1α-Cd4-His_6_ were injected over immobilized PEAR1 (*black lines*), and kinetic parameters for the interaction derived from a 1:1 binding model were fitted to the family of sensorgrams (*red lines*). *6H*, His_6_; *RU*, response units.

**Fig. 4.**
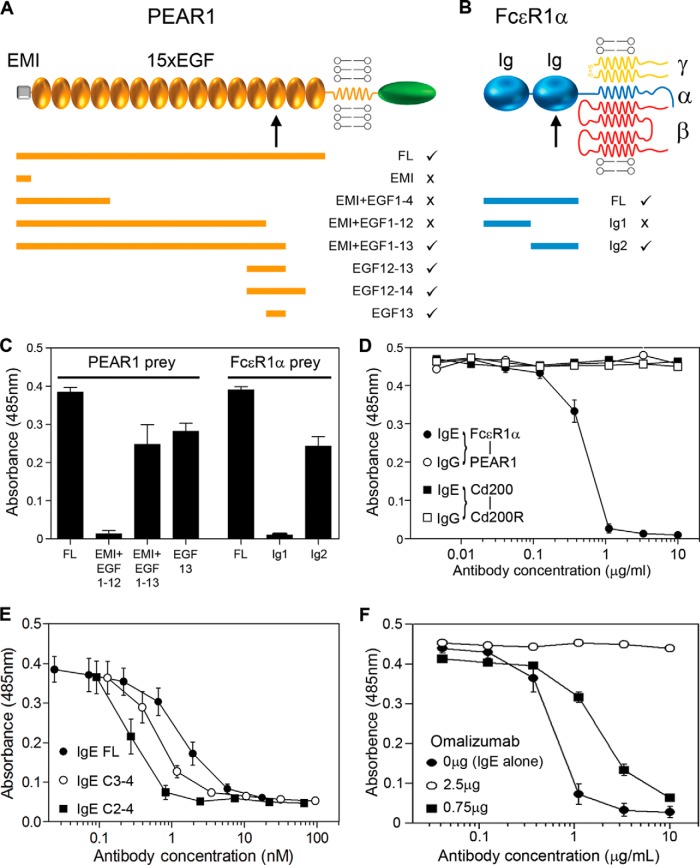
**The FcεR1α-PEAR1 interaction is mediated by membrane-proximal domains and can be specifically inhibited by IgE.** *A*, a schematic illustrating the domain organization of the PEAR1 receptor in the membrane. Expressed fragments of the PEAR1 protein are represented by *orange bars*, and the *ticks* and *crosses* indicate the ability of the fragments to bind the full-length (*FL*) ectodomain of FcεR1α as determined by AVEXIS; a similar summary for FcεR1α but tested for binding to PEAR1 is shown in *B. C*, binding data using AVEXIS showing that the 13th epidermal growth factor (*EGF*) domain of PEAR1 and the second Ig-like domain of FcεR1α are necessary and sufficient for binding. *Bars* represent means ± S.E. (*n* ≥ 3). *D*, the FcεR1α-PEAR1 interaction detected by AVEXIS using PEAR1 as a plate-immobilized bait was completely inhibited by low (IC_50_ ∼ 0.5 ng/ml) concentrations of IgE (*filled circles*) but not control IgG (*open circles*). A control interaction, rat Cd200-Cd200 receptor (*Cd200R*) (*squares*), was not inhibited by either antibody. *E*, the indicated concentrations of purified full-length (*FL*) and both smaller fragments of the IgE constant heavy chain (C2–4) and (C2–3) were preincubated with the FcεR1α prey before being added to the PEAR1 bait, and the interaction was detected using AVEXIS. Data points are mean ± S.E. (*n* = 3). *F*, the FcεR1α-PEAR1 interaction was detected using AVEXIS, and the inhibition by IgE (IgE alone; *filled circles*) could be relieved by the addition of 2.5 μg of omalizumab (*open circles*). Data points are mean ± S.E. (*n* ≥ 3). *Error bars* represent S.E. EMI, EMILIN-family domain.

To validate and quantify the interaction, we used surface plasmon resonance and observed clear saturable binding between PEAR1 and FcεR1α with an equilibrium binding constant (*K_D_*) of 27.4 ± 1.3 nm (Fig. 3*A*). The saturable binding behavior demonstrated the specificity of the interaction, which had a remarkably high affinity when compared with similar receptor-ligand interactions, which are usually in the micromolar range when measured using the same approach (10). An independent kinetic analysis confirmed this and revealed that the higher affinity was largely due to a comparatively slow dissociation rate constant (Fig. 3*B*). By using the AVEXIS assay and a series of structure-guided truncations in both PEAR1 and FcεR1α, we showed that the minimal FcεR1α binding unit on PEAR1 was contained solely within the 45 amino acids comprising the 13th epidermal growth factor domain (Fig. 4, *A* and *C*). Similarly, the PEAR1 binding site was located within the membrane-proximal Ig-like domain of the FcεR1α ectodomain, the same domain bound by IgE (27) (Fig. 4, *B* and *C*), raising the possibility that IgE may prevent PEAR1 binding when complexed with its receptor. Indeed, IgE, when bound to FcεR1α, could potently inhibit the FcεR1α-PEAR1 interaction at low concentrations, consistent with the high affinity of IgE for FcεR1α (Fig. 4*D*). To show that this inhibitory effect was due to blocking of the FcεR1α-PEAR1 interaction and not steric interference with the large (190-kDa) IgE molecule, we demonstrated that smaller subfragments of the IgE heavy chain that bound FcεR1α also blocked the FcεR1α-PEAR1 interaction (Fig. 4*E*). To demonstrate the specificity of this effect, we showed that addition of an anti-IgE monoclonal antibody that prevents IgE binding to FcεR1α (omalizumab) could relieve the inhibitory effect of IgE on the FcεR1α-PEAR1 interaction (Fig. 4*F*).

##### Oligomerized FcεR1α Specifically Potentiates Platelet Aggregation and Phosphorylates PEAR1

**Fig. 5.**
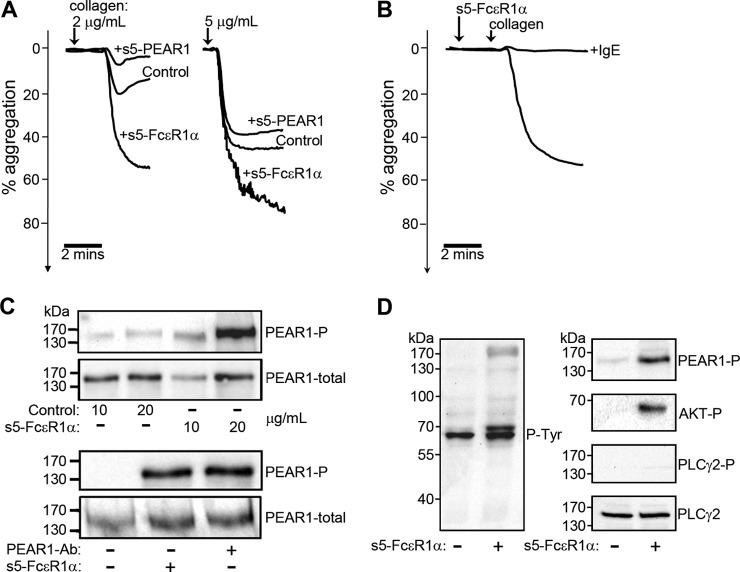
**Oligomerized FcεR1α promotes platelet aggregation and phosphorylates PEAR1.** *A*, soluble recombinant pentamerized (s5) PEAR1 ectodomains modestly inhibited whereas a similar s5-FcεR1α protein strongly promoted platelet aggregation relative to a control (rat s5-Cd200) when added prior to collagen-induced platelet aggregation. *B*, precomplexing s5-FcεR1α with IgE completely inhibited s5-FcεR1α-potentiated aggregation of collagen-activated platelets. *C*, oligomeric FcεR1α, but not a control protein, triggered the phosphorylation of PEAR1 (*top panel*) in human platelets with similar potency to an anti-PEAR1 antibody (*Ab*) (*lower panel*); total PEAR1 protein was detected as a loading control. *D*, oligomeric FcεR1α induced tyrosine phosphorylation in human platelets as shown by anti-phosphotyrosine (*P-Tyr*) Western blotting of lysates. Phosphorylation of PEAR1 and AKT but not PLCγ2 (a mediator of FcεR1α signaling) was observed. Total PLCγ2 protein was used as a loading control. Aggregation data are representative from at least 10 independent experiments.

To investigate the role of the FcεR1α-PEAR1 interaction in platelet activation, we confirmed cell surface expression of PEAR1 (8, 9) and FcεR1α (28, 29) by platelets using both Western blotting and immunocytochemistry (supplemental Fig. S5). Unlike PEAR1 (8), we did not observe any increase in the cell surface expression of FcεR1α after platelet activation. We next added clustered soluble oligomers (pentamers) of PEAR1 and FcεR1α to platelet aggregation assays and showed that they did not by themselves trigger platelet aggregation (data not shown). Preincubating unactivated platelets with FcεR1α oligomers followed by collagen stimulation, however, potentiated platelet aggregation (Fig. 5*A*). The specificity of this effect was demonstrated by precomplexing the FcεR1α pentamers with IgE to completely block FcεR1α-mediated PEAR1 signaling, suggesting the possibility that IgE could act as an endogenous plasma-borne restrictive regulator of thrombus formation (Fig. 5*B*). PEAR1 oligomers modestly inhibited platelet aggregation (Fig. 5*A*) and did not trigger known FcεR1α signaling effectors such as the phosphorylation of PLCγ (data not shown), suggesting that they competed with membrane-tethered PEAR1 for FcεR1α binding. Finally, oligomerized FcεR1α, but not a control, was able to trigger PEAR1 phosphorylation with similar potency to an activating anti-PEAR1 antibody (Fig. 5*C*) and led to clear AKT phosphorylation, a known mediator of PEAR1 signaling (8) (Fig. 5*D*).

## DISCUSSION

Platelets perform a delicately balanced role in hemostasis because they must detect and seal vascular breaches to restrict bleeding while ensuring a proportionate response to avoid vascular occlusion and maintain circulation. Interactions between receptor proteins displayed on the surface of platelets are a major class of thrombogenic regulator, and we report here a large recombinant protein library representing the secretome and receptor repertoire of the human platelet in a format suitable for systematic extracellular interaction screening using the AVEXIS assay. Importantly, we have expanded the utility of this assay to include receptor proteins from a greater range of structural classes as we work toward achieving a cell type- rather than a protein family-orientated screening approach; only the latter has been possible in the past (11–14, 24). Central to this approach is the use of a mammalian expression system to promote the correct folding of the receptor ectodomains, which we have previously shown can identify interactions that are functionally relevant *in vivo* (16–18). The human platelet protein library will be a useful resource in further defining the role of platelet receptors and their interactions in cardiovascular disease, particularly because all the expression plasmids have been made openly available through the Addgene repository (30).

We have demonstrated the usefulness of this resource by identifying the ligand for PEAR1, an “orphan” platelet receptor that is of topical interest because it has been identified in several recent independent genome-wide association studies linking it with variation in patients' responses to thrombogenic agonists in both health and disease (3–7). Because both FcεR1α and PEAR1 are expressed on the same cell, this raises the possibility that the two proteins might interact either in “*cis*” within the same membrane or in “*trans*” between neighboring cells. Although these need not be mutually exclusive, PEAR1 phosphorylation is known to be dependent upon platelet contact (8, 9) within forming aggregates, suggesting that the interaction is likely to occur in *trans*. The kinetic analysis of the interaction between the soluble monomeric proteins suggest that the two proteins interact with a 1:1 stoichiometry, although both proteins are likely to form signaling-competent clusters within the membrane, consistent with previous findings that bivalent, but not monovalent, anti-PEAR1 antibodies trigger PEAR1 phosphorylation (8). Soluble oligomers of FcεR1α, but not PEAR1, triggered known signaling effectors, consistent with a unidirectional signal triggered by the FcεR1α ligand through the PEAR1 receptor.

The function of the high affinity IgE receptor on platelets is poorly characterized, but previous work is consistent with the established role of IgE in immunity to parasitic infections because cross-linking FcεR1α on the platelet surface triggered platelet cytotoxicity to a parasitic worm, *Schistosoma mansoni* (29). Why FcεR1α has been adopted in the regulation of platelet biology is currently unclear, but perhaps the ability to block the interaction with endogenous plasma-borne IgE provides a clue. In healthy individuals, the concentration of circulating IgE is very low at ∼0.5 nm (31). Because the affinity of FcεR1α for IgE is within the same range (*K_D_* in the sub-nm range (32) and about 2 orders of magnitude higher than the affinity for PEAR1), this would suggest that in normal circulation a significant fraction, but not all (as shown in Hasegawa *et al.* (28)), of any FcεR1α on the surface of resting platelets will be bound by IgE and unable to interact with PEAR1. Where circulating levels of IgE are increased, for example in atopic patients (31), this would significantly decrease the amount of IgE-free FcεR1α on the platelet surface available for PEAR1 binding. This is consistent with reports of a systemic lack of secondary platelet responsiveness in atopic patients, an observation that in one study was correlated with elevated IgE levels (33–36). Others, however, have not replicated these findings (37, 38), suggesting a more complex relationship between circulating IgE levels and platelet function.

The controlled reduction of circulating IgE can be achieved in humans in the treatment of allergy with a humanized anti-IgE monoclonal antibody (omalizumab) that is currently licensed for the treatment of severe persistent allergic asthma (39). It is a systemic anti-IgE agent that prevents the interaction of IgE with its receptors, reducing plasma IgE levels by 99% and down-regulating FcεR1 on mast cells and basophils (40, 41). We have shown here that omalizumab is able to relieve the IgE-mediated inhibition of the FcεR1α-PEAR1 interaction, suggesting that omalizumab treatment could lead to alterations in the regulation of PEAR1 signaling. Indeed, concerns have recently been raised about an increased risk of arterial thrombotic events, particularly myocardial infarction and stroke, linked to the use of omalizumab (Ref. 42 and references therein). In conclusion, we believe that the platelet receptor protein microarray and plasmid resource will be a valuable tool in cardiovascular disease research, and the identification of FcεR1α as a ligand for PEAR1 makes an important contribution toward understanding the mechanistic role this receptor plays in platelet function and cardiovascular disease.

## Supplementary Material

Supplemental Data

## Footnotes

^1^ The abbreviations used are:

PEAR1: platelet endothelium aggregation receptor 1
AVEXIS: avidity-based extracellular protein interaction screen
FcεR1α: high affinity immunoglobulin E receptor subunit α
Ig: immunoglobulin
PLCγ: phospholipase Cγ
EC: extracellular domain
PEAR1-P: PEAR1 phosphorylation.
