# Supplementary material for: A Human Platelet Receptor Protein Microarray Identifies the High Affinity Immunoglobulin E Receptor Subunit α (FcεR1α) as an Activating Platelet Endothelium Aggregation Receptor 1 (PEAR1) Ligand
Source: Mol Cell Proteomics. 2015 Feb 23;14(5):1265–74. doi: 10.1074/mcp.M114.046946 (PMC4424398; doi:10.1074/mcp.M114.046946)
Supplement: Supplemental Data [file supp_14_5_1265__index.html]

A human platelet receptor protein microarray identifies FcεR1α as an activating PEAR1 ligand — A Human Platelet Receptor Protein Microarray Identifies the High Affinity Immunoglobulin E Receptor Subunit α (FcεR1α) as an Activating Platelet Endothelium Aggregation Receptor 1 (PEAR1) Ligand — FcεR1α Is a PEAR1 Ligand — Supplemental Data 

# A Human Platelet Receptor Protein Microarray Identifies the High Affinity Immunoglobulin E Receptor Subunit α (FcεR1α) as an Activating Platelet Endothelium Aggregation Receptor 1 (PEAR1) Ligand

## Supplemental Data

**Files in this Data Supplement:**

- Supplemental Information - Supplemental Information containing 5 supplementary figures and 2 supplementary tables.
