# Supplementary material for: A Human Platelet Receptor Protein Microarray Identifies the High Affinity Immunoglobulin E Receptor Subunit α (FcεR1α) as an Activating Platelet Endothelium Aggregation Receptor 1 (PEAR1) Ligand
Source: Mol Cell Proteomics. 2015 Feb 23;14(5):1265–74. doi: 10.1074/mcp.M114.046946 (PMC4424398; doi:10.1074/mcp.M114.046946)
Supplement: Supplemental Data [file supp_M114.046946_mcp.M114.046946-1.pdf]

Supplemental Information:

A human platelet receptor protein microarray identifies FcεR1α as an activating PEAR1 ligand

Yi Sun[1], Christophe Vandenbriele[2], Alexandre Kauskot[2], Peter Verhamme[2], Marc F. Hoylaerts[2] and Gavin J. Wright[1].

[1]Cell Surface Signalling Laboratory, Wellcome Trust Sanger Institute, Cambridge, CB10 1SA, United Kingdom.

[2]Center for Molecular and Vascular Biology, Department of Cardiovascular Sciences, University of Leuven, Leuven, Belgium.

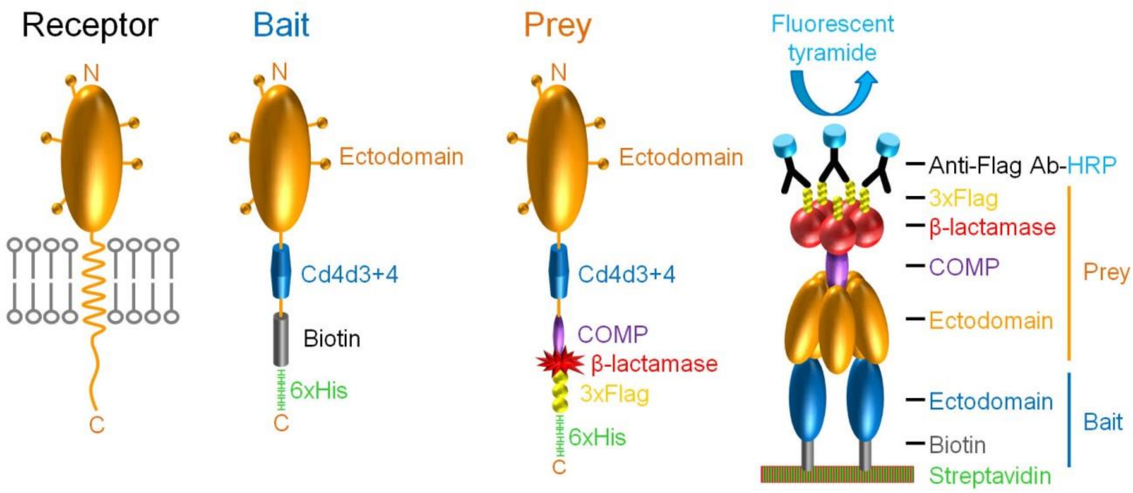

Supplemental Figure S1. Schematics showing design of bait and prey constructs and the AVEXIS interaction assay. A typical type I cell surface receptor is shown embedded within a membrane with the entire ectodomain shown as an orange oval, and filled lollipops representing potential N-linked glycosylation sites. The design of both monomeric biotinylated "bait" and pentameric β-lactamase-tagged "prey" containing the entire ectodomain of the receptor are shown (prey is shown as a monomer for clarity); both contain the rat Cd4 domain 3 and 4 (Cd4d3+4) and 6 His tags. The AVEXIS assay is shown with a biotinylated bait captured on streptavidin-coated slide interacting with a pentameric FLAG-tagged prey and interactions detected with an anti-FLAG HRP-conjugated secondary antibody and fluorescent tyramide HRP-substrate deposition.

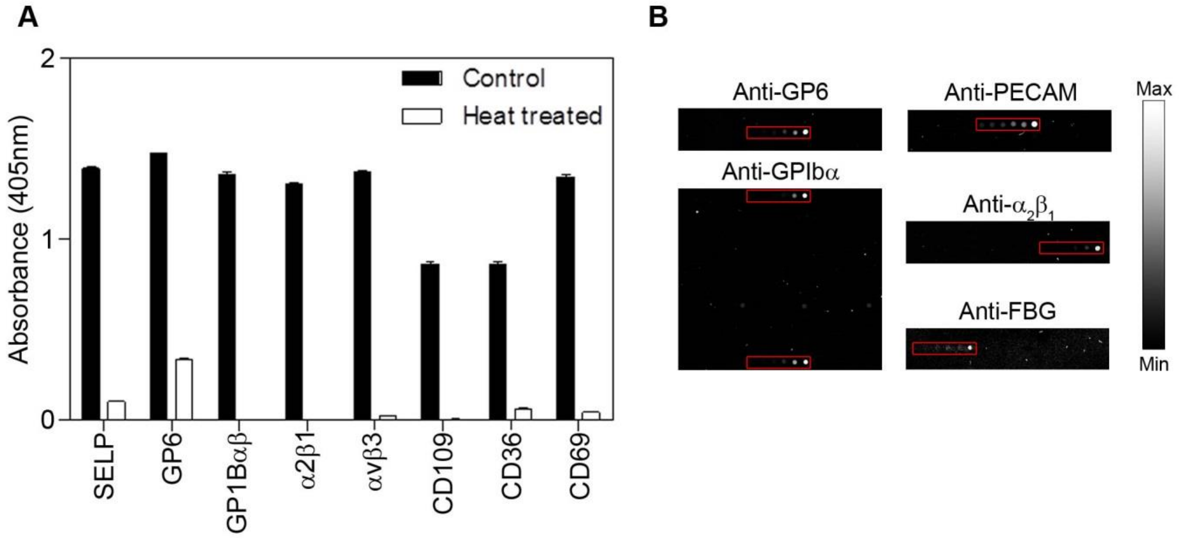

Supplemental Figure S2. Proteins in the human platelet receptor library are antigenically active. (A) The biochemical activity of selected recombinant proteins representing each structural class was confirmed by demonstrating heat-labile immunoreactivity of monoclonal antibodies which are known to stain the native protein on the platelet surface. The heat-treated and control proteins were probed with monoclonal antibodies (SELP: Thromb6; GP6: HY101; GP1Bαβ: PAB-5; α2β1: P1E6; αvβ3: BV3; CD109: B-E47; CD36: CB38; CD69: FN50) and detected by ELISA. Bars represent mean ± SEM; $n \geq 3$. (**B**) Immunoreactivity to mAbs recognizing the individual named platelet receptors that are known to bind native proteins on platelets is preserved when arrayed on streptavidin-coated glass slides; note that anti-GP1bα identifies both the GP1bα monomer (bait 75) and GP1bαβ complex (bait 77).

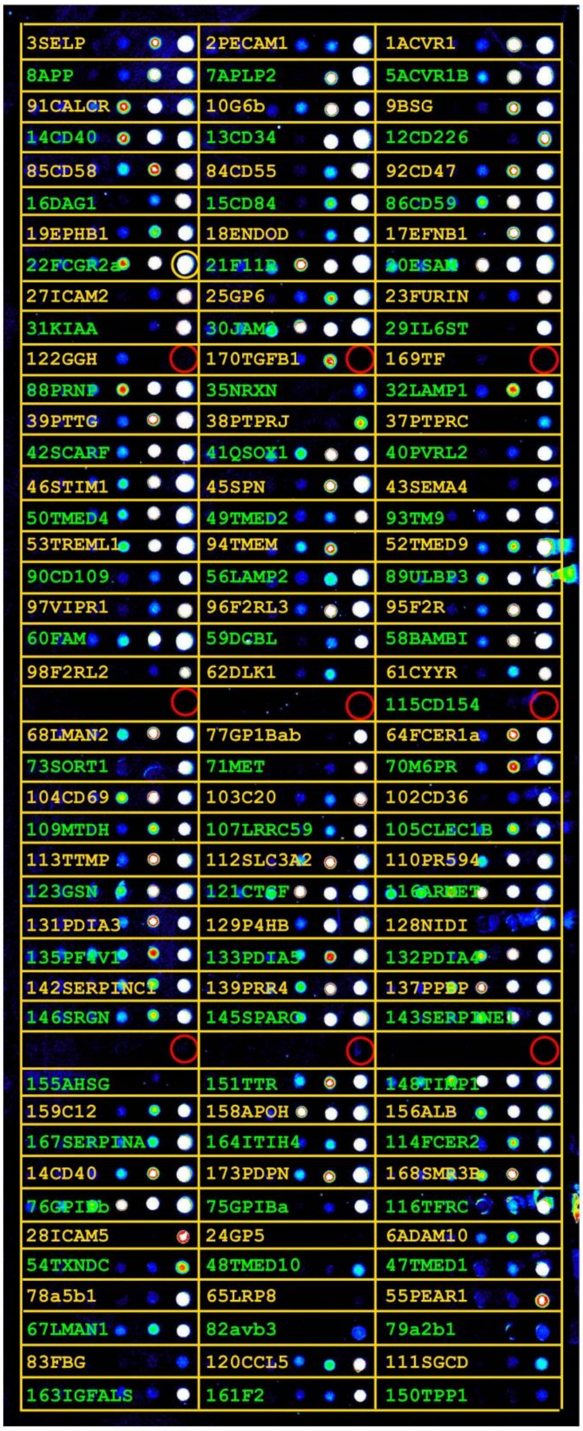

Supplemental Figure S3. Map of the human platelet receptor protein microarray. The image shows the platelet receptor microarray containing biotinylated ectodomains of human platelets receptor "baits" immobilized on a streptavidin-coated slide. The array was stained with a mouse anti-rat Cd4 mAb common to all baits, followed by an anti-mouse Cy3-conjugated secondary. The area containing the dilutions of each bait is boxed and numbered/described according to Supplemental Table 2. The highest dilution of the bait containing the IgG Fc receptor, FCGR2a (bait 22), which interacts directly with the HRP-conjugated anti-FLAG secondary antibody in the AVEXIS protocol, is circled in yellow. The positions of nine spots of biotinylated HRP that are arrayed as orientation markers, which are not stained in this experiment, are indicated with red circles.

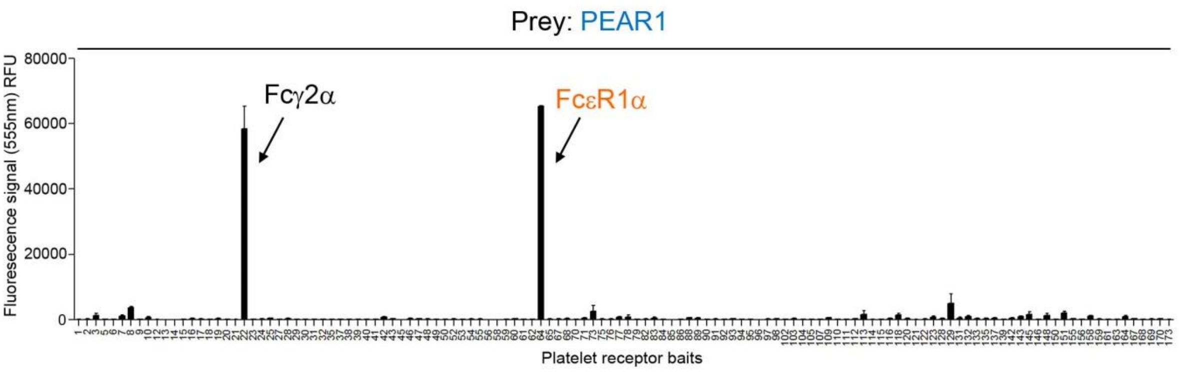

Supplemental Figure S4. Systematic screening of a human platelet receptor protein microarray identified FcεR1α as a ligand for PEAR1: quantification of fluorescence signals. Quantification of the florescence at 555 nm associated with each bait on a human platelet receptor protein microarray probed with FLAG-tagged PEAR1 prey protein identifying FcεR1α (bait 64) as a ligand for PEAR1. The IgG receptor, Fcγ2α (bait 22) directly interacted with the HRP-conjugated secondary antibody. Baits are numbered as described in Supplementary Table 2; bars represent mean florescence signals ± SEM; *n* = 3.

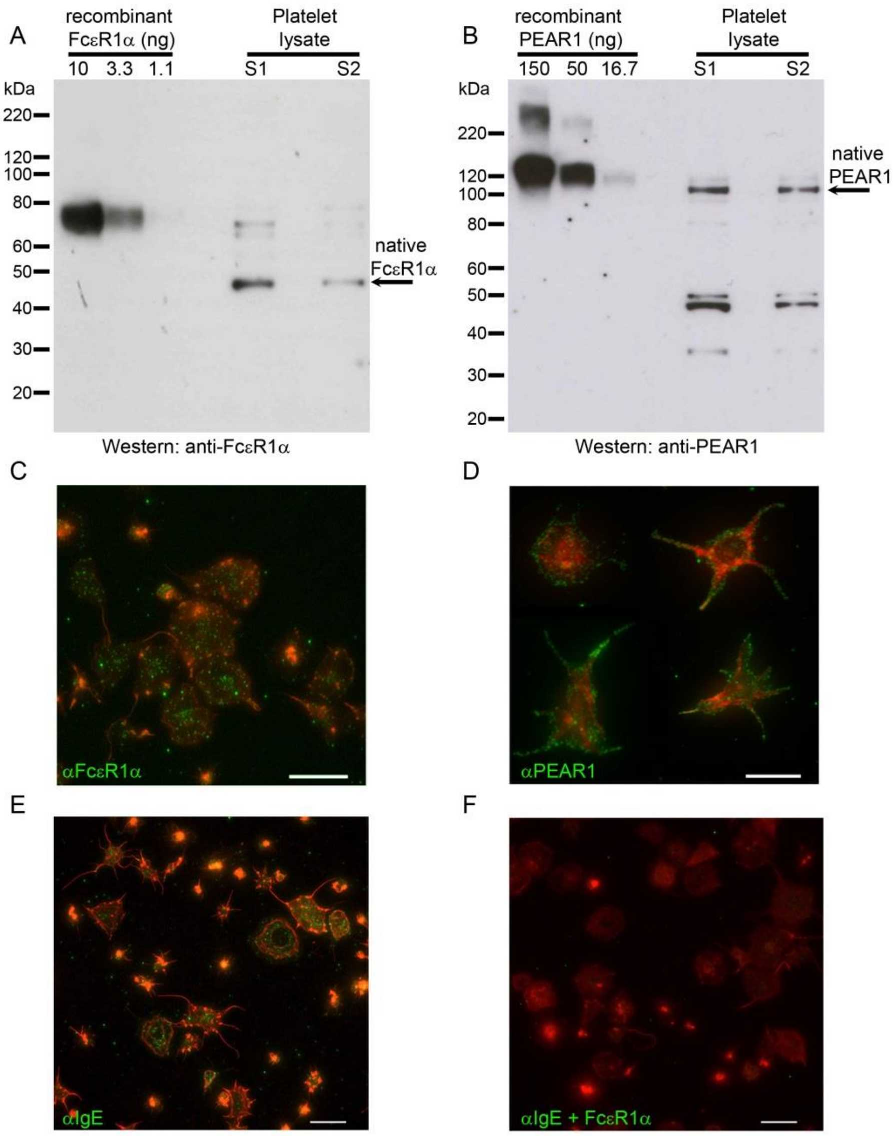

Supplemental Figure S5. FcεR1α and PEAR1 are both expressed by human platelets. (**A**, **B**) Western blot analysis showing gels loaded with the indicated amount of recombinant monomeric FcεR1α (**A**) and PEAR1 (**B**) and platelet lysate samples from two individuals (S1 and S2, at 1.95×10^7 platelet equivalents per lane) probed with antibodies raised against the extracellular regions of human FcεR1α and PEAR1. Surface expression of both PEAR1 and FcεR1α was confirmed by immunofluorescence antibody staining on platelets (data not shown). Note that the molecular mass difference between the recombinant FcεR1α and PEAR1 proteins versus the native proteins is due to the tags present in the recombinant proteins. Blots shown are representative of three independent experiments. (**C**-**F**) Staining of platelets spread over a fibrinogen matrix and stained with either anti-FcεR1α (**C**) or anti-PEAR1 (**D**) in green and counter-stained with phalloidin (red). (**E**-**F**) Platelets were spread over a fibrinogen matrix and stained with anti-IgE (green). The IgE staining pattern was punctate (**E**) and shown to be both specific and extracellular since it could be abolished by incubating the platelets with an excess of soluble recombinant FcεR1α (**F**). Unlike PEAR1, cell surface FcεR1α is not increased upon platelet activation and using densitometry and the recombinant protein standards, we estimate that FcεR1α is expressed at ~10-fold lower levels than PEAR1 in platelets. Scale bars represent 5 µm.

Supplemental Table S1

	A list of proteomics-based studies to identify proteins expressed by human platelets	
Number	Descriptions	Ref.
1	Mainly cytosolic fraction, tyrosine phosphorylated proteins	Marcus K. et al., Electrophoresis 21, 2622 (2000)
2	Thrombin activated platelets, total phosphotyrosine proteome	Maguire P. B. et al., Proteomics 2, 642 (2002)
3	Total cellular proteins	O'Neill E. E. et al., Proteomics 2, 288 (2002)
4	Cytosolic and membrane fractions	Gevaert K. et al., Nat Biotechnol 21, 566 (2003)
5	Activated platelet releasates	Coppinger J. A. et al., Blood 103, 2096 (2004)
6	Total cellular proteins	Garcia A. et al., Proteomics 4, 656 (2004)
7	Activated platelet, total cellular proteins	Garcia A. et al., Blood 103, 2088 (2004)
8	Activated platelet, secreted proteins	McRedmond J. P. et al., Mol Cell Proteomics 3, 133 (2004)
9	Cytosolic and membrane fractions, multimeric proteins	Claeys D. et al., Electrophoresis 26, 1189 (2005)
10	Total cellular proteins	Martens L. et al., Proteomics 5, 3193 (2005)
11	Membrane proteins	Moebius J. et al., Mol Cell Proteomics 4, 1754 (2005)
12	Activated platelets, tyrosine phosphorylated proteins	Garcia A. et al., Proteomics 6, 5332 (2006)
13	Enriched with low-abundance proteins	Guerrier L. et al., J Proteome Res 6, 4290 (2007)
14	Dense granule proteins	Hernandez-Ruiz L. et al., J Proteome Res 6, 4449 (2007)
15	Plasma membrane proteins	Lewandrowski U. et al., Mol Cell Proteomics 6, 1933 (2007)
16	α-granule proteins	Maynard D. M. et al., J Thromb Haemost 5, 1945 (2007)
17	Plasma membrane and intracellular membrane proteins	Senis Y. A. et al., Mol Cell Proteomics 6, 548 (2007)
18	Resting platelets, phosphoproteome	Zahedi R. P. et al., J Proteome Res 7, 526 (2008)

Supplemental Table S2. The human platelet ectodomain protein library. Listed for each protein within the library is: the abbreviation of the protein, UniProt accession number, ectodomain truncation residue (Trun.), bait vector used to clone the ectodomain (BLH = Bio-linker-His vector, HLB = His-linker-Bio vector), and bait expression level (mg/ml) where "0" means no detectable protein expression.

Number	Proteins	UniProt	Trun.	Bait Vector	Bait Expression (mg/ml)
	Type 1, single ectodomain				
**1**	ACVR1	Q04771	G125	BLH	10
**2**	PECAM1	P16284	G602	BLH	1.5
**3**	SELP	P16109	A771	BLH	1.1
**4**	C1QBP	Q07021	Q282	BLH	0
**5**	ACVR1B	P36896	P124	BLH	10
**6**	ADAM10	O14672	W673	BLH	0.687
**7**	APLP2	Q06481	S693	BLH	3.6
**8**	APP	P05067	G700	BLH	1.2
**9**	BSG	P35613	L322	BLH	5
**10**	G6B	O95866	P141	BLH	1.2
**11**	CCDC	Q8NE86	T233	BLH	0
**12**	CD226	Q15762	F252	BLH	2
**13**	CD34	P28906	T290	BLH	3.6
**14**	CD40	P25942	L192	BLH	10
**15**	CD84	Q9UIB8	T224	BLH	1.2
**16**	DAG1	Q14118	T753	BLH	2
**17**	EFNB1	P98172	L240	BLH	3.6
**18**	ENDOD	O94919	P540	BLH	3.8
**19**	EPHB1	P54762	P245	BLH	3.5
**20**	ESAM	Q96AP7	P245	BLH	3.6
**21**	F11R	Q9Y624	G237	BLH	5
**22**	FCGR2a	P12318	P215	BLH	4
**23**	FURIN	P09958	P714	BLH	0.98
**24**	GP5	P40197	P520	BLH	0.113
**25**	GP6	Q9HCN6	G268	BLH	0.8
**26**	GP9	P14770	P146	BLH	0
**27**	ICAM2	P13598	M224	BLH	1
**28**	ICAM5	Q9UMF0	P834	BLH	0.245
**29**	IL6ST	P40189	E619	BLH	1.2
**30**	JAM3	Q9BX67	G243	BLH	10
**31**	KIAA	Q14165	S270	BLH	1.2
**32**	LAMP1	P11279	P385	BLH	5

Number	Proteins	UniProt	Trun.	Bait Vector	Bait Expression (mg/ml)
	Type 1, single ectodomain				
**33**	LRRC	Q14392	N627	BLH	0
**34**	NPHS	O60500	P1064	BLH	0
**35**	NRXN	Q9ULB1	T1401	BLH	2.4
**36**	PCDH	Q9Y5H9	N697	BLH	0
**37**	PTPRC	P08575	L577	BLH	1.7
**38**	PTPRJ	Q12913	P970	BLH	1.8
**39**	PTTG	P53801	E96	BLH	8.5
**40**	PVRL2	Q92692	G359	BLH	1
**41**	QSOX1	O00391	G703	BLH	6.7
**42**	SCARF	Q14162	T421	BLH	0.81
**43**	SEMA4	Q92854	S707	BLH	6.3
**44**	SIGLE	Q96PQ1	T478	BLH	0
**45**	SPN	P16150	P257	BLH	3.6
**46**	STIM1	Q13586	D213	BLH	10
**47**	TMED1	Q13445	R192	BLH	0.69
**48**	TMED10	P49755	R185	BLH	0.166
**49**	TMED2	Q15363	R168	BLH	2.2
**50**	TMED4	Q7Z7H5	R194	BLH	3.6
**51**	TMED5	Q9Y3A6	R194	BLH	0
**52**	TMED9	Q9BVK6	R181	BLH	3.6
**53**	TREML1	Q86YW5	P162	BLH	10
**54**	TXNDC	Q9H3N1	P137	BLH	0.3
**55**	PEAR1	Q5VY43	S754	BLH	0.52
**56**	LAMP2	P13473	P378	BLH	3.6
**57**	MPL	P40238	S489	BLH	0
**58**	BAMBI	Q13145	S145	BLH	12
**59**	DCBL	Q96PD2	P475	BLH	10
**60**	FAM	Q5VUB5	T300	BLH	4.8
**61**	CYYR	Q96J86	T61	BLH	5.4
**62**	DLK1	P80370	A304	BLH	1.5
**63**	EGF	P01133	K1032	BLH	0
**64**	FCER1a	P12319	K201	BLH	5

Number	Proteins	UniProt	Trun.	Bait Vector	Bait Expression (mg/ml)
	Type 1, single ectodomain				
**65**	LRP8	Q14114	T824	BLH	0.142
**66**	INSR	P06213	K956	BLH	0
**67**	LMAN1	P49257	T478	BLH	0.73
**68**	LMAN2	Q12907	G320	BLH	10
**69**	G6f	Q5SQ64	K208	BLH	0
**70**	M6PR	P20645	S187	BLH	1.1
**71**	MET	P08581	P927	BLH	2
**72**	NOTCH3	Q9UM47	R1570	BLH	0
**73**	SORT1	Q99523	P758	BLH	1.6
**74**	CD44	P16070	E649	BLH	0
**75**	GP1BAm	P07359	P497	BLH	2
**76**	GP1BBm	P13224	P145	BLH	3.5
	Multi-subunits proteins				
**77**	GP1Bαβ	P07359/P13224	L502/W148	BLH, α-chain tagged	1.2
**78**	ITGA5/ITGB1	P08648/P05556	P998/P727	BLH, α-chain tagged	0.55
**79**	ITGA2/ITGB1	P17301/P05556	P1131/P727	BLH, α-chain tagged	0.32
**80**	ITGA6/ITGB1	P23229/P05556	P1053/P727	BLH, α-chain tagged	0
**81**	ITGA2B/ITGB3	P08514/P05106	P996/P717	BLH, α-chain tagged	0
**82**	ITGAV/ITGB3	P06756/P05106	P993/P717	BLH, α-chain tagged	0.38
**83**	FBG	P02671/P02675/P02679	Q866/Q491/L453	BLH, α-chain tagged	0.1
	GPI-anchored proteins				
**84**	CD55	P08174	S353	BLH	1.4
**85**	CD58	P19256	R215	BLH	10
**86**	CD59	P13987	G103	BLH	10
**87**	OTOA	Q7RTW8	P1129	BLH	0
**88**	PRNP	P04156	S230	BLH	3.6
**89**	ULBP3	Q9BZM4	P216	BLH	3.6
**90**	CD109	Q6YHK3	S1423	BLH	0.83

Number	Proteins	UniProt	Trun.	Bait Vector	Bait Expression (mg/ml)
	Multi-transmembrane proteins with a long extracellular domain				
**91**	CALCR	P30988	Y150	BLH	3.6
**92**	CD47	Q08722	P139	BLH	3
**93**	TM9	Q92544	H279	BLH	10
**94**	TMEM	Q9BVC6	S82	BLH	10
**95**	F2R	P25116	P106	BLH	10
**96**	F2RL3	Q96RI0	T77	BLH	15
**97**	VIPR1	P32241	G145	BLH	1.2
**98**	F2RL2	O00254	P98	BLH	2.1
**99**	ENTP	P49961	T38-T478	BLH	0
**100**	P2RX1	P51575	E52-S337	BLH	0
**101**	P2RX6	O15547	A60-P336	BLH	0
**102**	CD36	P16671	G30-N439	BLH	1.2
	Type 2 proteins				
**103**	C20	Q9HDC9	E62-V416	HLB	1.4
**104**	CD69	Q07108	P69-K199	HLB	0.8
**105**	CLEC1B	Q9P126	G52-P229	HLB	0.75
**106**	ECE1	P42892	G88-W770	HLB	0
**107**	LRRC59	Q96AG4	C266-Q307	HLB	7.2
**108**	LRRC8b	Q6P9F7	G327-C803	HLB	0
**109**	MTDH	Q86UE4	S84-T582	HLB	8.8
**110**	PR594	P42857	K104-A185	HLB	3
**111**	SGCD	Q92629	K57-L289	HLB	0.169
**112**	SLC3A2	P08195	R211-A630	HLB	10
**113**	TTMP	Q5BVD1	A89-E217	HLB	5.6
**114**	FCER2	P06734	D48-S321	HLB	8
**115**	CD154	P29965	H47-L261	HLB	0.03
**116**	TFRC	P02786	C89-F760	HLB	10

Number	Proteins	UniProt	Trun.	Bait Vector	Bait Expression (mg/ml)
	Secreted				
**117**	ANGPT	Q15389	F498	BLH	0
**118**	ARMET	P55145	L179	BLH	7
**119**	C21	P30042	K268	BLH	0
**120**	CCL5	P13501	S91	BLH	0.26
**121**	CTGF	P29279	A349	BLH	1.2
**122**	GGH	Q92820	D318	BLH	0.07
**123**	GSN	P06396	A482	BLH	6
**124**	HINT2	Q9BX68	G163	BLH	0
**125**	HPSE	Q9Y251	I543	BLH	0
**126**	MMP11	P24347	L488	BLH	0
**127**	MMRN1	Q13201	T1228	BLH	0.01
**128**	NID1	P14543	K1247	BLH	5.6
**129**	P4HB	P07237	L508	BLH	4.9
**130**	PCYOX1L	Q8NBM8	L494	BLH	0
**131**	PDIA3	P30101	L505	BLH	1.6
**132**	PDIA4	P13667	L645	BLH	6.1
**133**	PDIA5	Q14554	L519	BLH	2.5
**134**	PF4	P02776	S101	BLH	0.01
**135**	PF4V1	P10720	S104	BLH	4.5
**136**	PLOD2	O00469	P737	BLH	0
**137**	PPBP	P02775	D128	BLH	1
**138**	PPIB	P23284	E216	BLH	0.01
**139**	PRR4	Q16378	W134	BLH	0.92
**140**	PTGS1	P23219	L599	BLH	0
**141**	SCUBE1	Q8IWY4	K988	BLH	0
**142**	SERPINC1	P01008	K464	BLH	1.2
**143**	SERPINE1	P05121	P402	BLH	6.4
**144**	SERPINE2	P07093	P398	BLH	0
**145**	SPARC	P09486	I303	BLH	10

Number	Proteins	UniProt	Trun.	Bait Vector	Bait Expression (mg/ml)
	Secreted				
**146**	SRGN	P10124	L158	BLH	1
**147**	THBS1	P07996	P1170	BLH	0
**148**	TIMP1	P01033	A207	BLH	12
**149**	TIMP3	P35625	P211	BLH	0
**150**	TPP1	O14773	P563	BLH	0.18
**151**	TTR	P02766	E147	BLH	1.2
**152**	VWF	P04275	K2813	BLH	0.003
**153**	LTBP1	Q14766	E1721	BLH	0
**154**	A2M	P01023	A1474	BLH	0
**155**	AHSG	P02765	V367	BLH	10
**156**	ALB	P02768	L609	BLH	10
**157**	APCS	P02743	V223	BLH	0
**158**	APOH	P02749	C345	BLH	1.5
**159**	C12	Q9BT56	W116	BLH	2
**160**	ECM2	O94769	K699	BLH	0
**161**	F2	P00734	E622	BLH	0.33
**162**	F5	P12259	Y2224	BLH	0
**163**	IGFALS	P35858	C605	BLH	0.27
**164**	ITIH4	Q14624	L930	BLH	1.4
**165**	PLG	P00747	N810	BLH	0
**166**	PLUNC	Q9NP55	V256	BLH	0
**167**	SERPINA	P01009	K418	BLH	2.5
**168**	SMR3B	P02814	P79	BLH	1
**169**	TF	P02787	P698	BLH	0.28
**170**	TGFB1	P01137	S390	BLH	0.46
**171**	VTN	P04004	L478	BLH	0.01
**172**	WNT11	O96014	K354	BLH	0
**173**	PDPN	Q86YL7	L131	BLH	4.5
